# Retrospective evaluation of trained and untrained probabilistic ensemble forecasts for influenza hospital admissions — United States, 2022–2025

**DOI:** 10.1016/j.idm.2026.04.002

**Published:** 2026-04-06

**Authors:** Aaron M. Frutos, Annabella Hines, Li Shandross, Evan L. Ray, Nicholas G. Reich, Rebecca K. Borchering, Matthew Biggerstaff

**Affiliations:** a Influenza Division, National Center for Immunization and Respiratory Diseases, Centers for Disease Control and Prevention, United States; b Epidemic Intelligence Service, Centers for Disease Control and Prevention, United States; c STI Federal, Chippewa Government Solutions, United States; d School of Public Health and Health Sciences, University of Massachusetts Amherst, United States

**Keywords:** Influenza, Forecasting, Ensemble

## Abstract

The United States Centers for Disease Control and Prevention (CDC) coordinates influenza forecasting efforts with approximately 30 academic and industry teams and combines their short-term, weekly forecasts into an ensemble forecast to improve accuracy and increase utility. To investigate the accuracy of trained ensemble methods for forecasting confirmed influenza hospital admissions, we retrospectively compared ensembles trained on past forecast performance of submitting teams during the 2022–23, 2023–24, and 2024–25 influenza seasons to the untrained ensemble used during each season. Forecasts are based on laboratory-confirmed influenza hospital admission data from CDC's National Healthcare Safety Network. For each week from October 2022–April 2023, October 2023–April 2024, and November 2024–May 2025, we produced three trained median and three trained mean ensembles that weight individual forecasts based on their weighted interval score (WIS) during the prior 2, 4, or 6 weeks of performance. We evaluated the trained and untrained ensembles using prediction interval coverage and the WIS. Compared to the untrained ensemble, multiple trained ensemble performed better in each season and across jurisdictions, although the best performing ensemble differed. As this is an analysis of only three influenza seasons, we will continue to evaluate ensemble performance over subsequent seasons to see if consistent patterns emerge in the performance of different methods to train ensembles.

## 1 Introduction

Accurate influenza hospitalization predictions provide public health officials with situationally relevant information to guide influenza prevention and control initiatives. From 2013 until 2026 (the time of writing), the United States Centers for Disease Control and Prevention (CDC) has coordinated influenza forecasting efforts (About Flu Forecasting, 2024). In the 2024–25 season, approximately 30 academic and industry partners submitted forecasts of weekly influenza hospital admissions. CDC combined these short term-forecasts into an untrained probabilistic ensemble forecast to improve accuracy.

During the COVID-19 pandemic, the United States COVID-19 Forecast Hub, used by CDC to inform decision making, investigated the use of trained probabilistic ensembles for predicting COVID-19 cases and deaths (Ray et al., 2023). They found that trained ensembles did well when individual component forecasts had good and stable performance (e.g., COVID-19 death forecasts) but were less accurate when component forecaster skill varied week to week (e.g., COVID-19 case forecasts).

To investigate the utility of a trained ensemble for forecasts of influenza hospitalizations, we retrospectively compared the untrained ensembles used during the 2022–23, 2023–24, and 2024–25 influenza seasons to trained ensembles that weighted individual models based on the recent past forecast performance of their submitted forecasts.

## 2 Material and methods

During the 2022–23 and 2023–24 seasons, academic and industry partners submitted weekly influenza hospital admissions forecasts from October–April based on influenza hospital admissions data from CDC's National Healthcare Safety Network (NHSN) (Hospital Respiratory Data, 2024; Mathis et al., 2024). For the 2024–25 season, the submission period was November through May. In the 2022–23 season, 26 teams submitted forecasts, followed by 27 teams in the 2023-24 season, and 30 in the 2024-25 season. NHSN includes data from all hospitals registered with the Centers for Medicare and Medicaid. Submitted forecasts include national- and jurisdiction-specific quantile predictions of weekly laboratory-confirmed influenza hospital admissions which are made publicly available online (Flusight-forecast-data, 2023; FluSight-forecast-hub, 2024). Forecasts were made for the current week and 1, 2, and 3 weeks after the forecast date.

### 2.1 Creating weighted ensemble forecasts

We created trained ensembles from these submitted component forecasts applying a similar approach as the United Stated COVID-19 Forecast Hub (Ray et al., 2023) using the hubEnsembles R package (Shandross et al., 2026). Submitted component forecasts were evaluated using the weighted interval score (WIS) (Bracher et al., 2021) and the scoringutils R package (scoringutils, 2023) based on data available at the time the forecast was submitted; here relative WIS (rWIS) (the ratio of the mean WISs for two models across all horizons, all locations and all forecast dates during the same period) (Cramer et al., 2022) was calculated with respect to a baseline forecast, which was produced using the previous week's observations with noise generated using positive and negative 1-week differences (Mathis et al., 2024). We used a training window of 2, 4, and 6 weeks for median and for mean trained ensembles. We trained and weighted a separate ensemble for the total number of hospital admissions in the US and an ensemble for the total number of hospital admissions by state and territory; we produced these two ensembles because of the difference in the number of forecasts submitted for total hospital admissions in the United States and those submitted by state and territory. We calculated weights (w) as a sigmoidal transformation of the forecasts' rWIS using the following formula where a component forecast is denoted by m, forecast date by s, and the total number of available forecasts by M (Ray et al., 2023):

$$w_{s}^{m}=\frac{\exp\left(-\theta_{s}\cdot {rWIS}_{s}^{m}\right)}{\sum_{m^{\prime }=1}^{M}\exp\left(-\theta_{s}\cdot {rWIS}_{s}^{m^{\prime }}\right)}$$

We implemented a grid search to select the value of $\theta_{s}$ that would optimize the WIS of the ensemble forecast during the training window (the preceding 2, 4, or 6 weeks). To limit the contribution of a given component forecast, we used a weight limit of 0.3 (Ray et al., 2023).

We used forecasts from October 17, 2022–February 27, 2023, October 14, 2023–April 27, 2024, and November 22, 2024–May 31, 2025, to generate weighted ensembles and compared these to corresponding unweighted ensembles. We limited the included forecasts from the 2022–2023 season from October to February because influenza virus spread was atypically early that season and influenza hospital admissions were at low levels by the end of February (Influenza Activity in the United States, 2023). To include the same number of forecasts for ensembles with different training periods, we generated ensembles after component forecasts had been submitted for at least 6 weeks each season. To be included in the weighted ensembles for a given week, component forecasts must have had a forecast for that week and every week of the training period. For the 2024-25 season, submissions were paused during the week of January 25, 2025; any training period that included that week instead used forecasts from the six most recent weeks with available forecasts.

### 2.2 Evaluating weighted and unweighted ensemble forecasts

We used weighted interval score (WIS), relative weighted interval score (rWIS) (relative to the WIS of the unweighted median ensemble), mean absolute error (MAE), and prediction interval coverage (50% and 95%) to evaluate the performance of the ensembles based on reported data available on July 19, 2025. WIS is a forecast skill metric that measures how consistent a group of forecast prediction intervals is with observed data. For evaluating the trained ensembles, we used the unweighted median ensemble as the comparator model to calculate the rWIS, as it is the current operational ensemble used in FluSight and has demonstrated robust performance across prior influenza forecasting efforts (Centers for Disease Control and Prevention, 2025a, Centers for Disease Control and Prevention, 2025b). In this case, weighted ensembles that perform better than the unweighted median ensemble would have an rWIS values < 1, while weighted ensembles with worse performance than the unweighted median ensemble would have rWIS values > 1. MAE quantifies the average magnitude of errors in a set of predictions. Coverage values indicate how often the prediction interval (e.g., 50%) contained the eventually observed value. Ideally, coverage values are the same as the prediction intervals they correspond to, i.e., a 50% prediction interval should contain the ultimately observed value 50% of the time. These forecast scoring metrics have been described previously (Mathis et al., 2024). Due to a pause in mandatory hospital reporting to NHSN on May 1, 2024, ensemble forecasts with targets past that point were not included in scoring.

## 3 Results

**Fig. 1:**
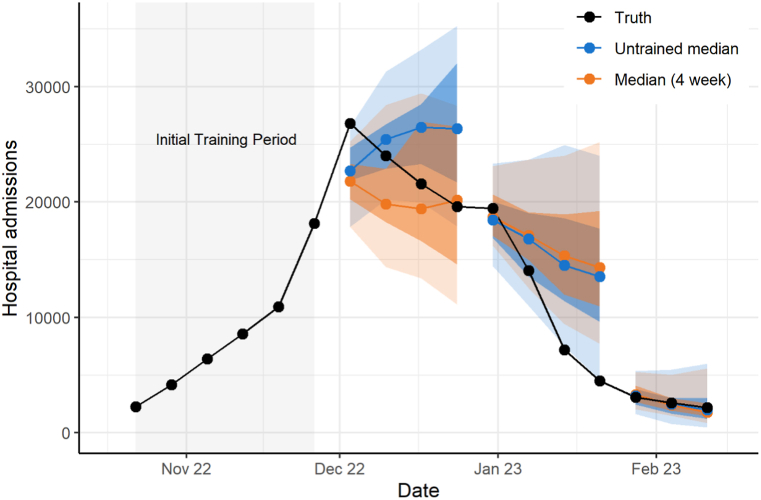
**National weekly observed hospital admissions and forecast distribution with 50% and 95% prediction intervals for the top performing trained ensemble (4-week median) and untrained ensembles during the 2022**–**23 influenza season.** We show each horizon (current and up to three weeks after) for each forecast. To avoid plotting overlapping forecasts, we only display ensemble forecasts every four weeks. The median ensemble forecast values (colored points) are shown with the corresponding 50% and 95% prediction intervals (colored shaded regions). Truth data were observed as of July 19, 2025.

For the 2022–2023 season, we used 26 different component forecasts to produce trained ensembles for 14 weeks. For a given week, the number of forecasts included ranged from 10 to 21 for the national ensemble and 4 to 9 for the state/territory ensemble; component forecast weight distribution varied throughout the season (Supplemental Fig. 1). For the national ensemble, the median trained ensemble with a 4- or 6-week training window had a rWIS of 0.97, indicating slightly better performance than the untrained median (Table 1) while all mean trained ensembles had higher WIS (indicating worse performance) than the untrained median and the baseline. Trained ensembles could demonstrate worse performance than untrained ensembles if the best performing models change through time, for example. No trained national ensemble during the 2022–2023 season had 95% prediction interval coverage values closer to 95% than the untrained ensemble. There was no obvious pattern as to when the trained or untrained forecasts were closer to the observed reported number of influenza hospital admissions (Fig. 1). For the state/territory ensemble, all median and mean trained ensembles performed better than the untrained median, and the trained median with a 2-week training window performed the best (rWIS of 0.87); all trained state/territory ensembles also had 95% coverage that was closer to 95% than that of the untrained median.

**Table 1:** **Trained ensemble performance at the national and state/territory level for the 2022**–**23, 2023**–**24, and 2024**–**25 influenza seasons.** Ensembles from the 2022–23 season were created using forecasts from October 17, 2022–February 27, 2023. Ensembles from the 2023–24 season were created using forecasts from October 14, 2023 –April 27, 2024. Ensembles from the 2024–25 season were created using forecasts from November 22, 2024–May 31, 2025. rWIS is calculated relative to the untrained median.

Model (training period)	WIS	rWIS	MAE	50% PI Coverage (in %)	95% PI Coverage (in %)
**National Ensemble, 22**–**23**					
Median (4 week)	2502	0.97	3579	57.7	78.8
Median (6 week)	2503	0.97	3586	61.5	73.1
Untrained Median∗	2590	1.00	3931	57.7	80.8
Median (2 week)	2851	1.10	4144	46.1	78.8
Baseline	3716	1.43	4649	32.7	63.5
Mean (2 week)	6018	2.32	5669	40.4	75.0
Mean (4 week)	9145	3.53	5340	55.8	78.8
Mean (6 week)	9301	3.59	5453	59.6	71.2
**National Ensemble, 23**–**24**					
Median (6 week)	1650	0.91	2755	39.5	93.0
Mean (6 week)	1762	0.98	2883	34.9	94.2
Median (2 week)	1788	0.99	2794	46.5	90.7
Untrained Median∗	1804	1.00	2890	44.2	90.7
Median (2 week)	1847	1.02	2879	39.5	89.5
Mean (4 week)	1896	1.05	2938	44.2	93.0
Median (4 week)	2005	1.11	2939	44.2	90.7
Baseline	2454	1.36	3369	10.5	84.9
**National Ensemble, 24**–**25**					
Mean (2 week)	2272	0.80	3139	76.2	96.4
Mean (4 week)	2310	0.82	3317	76.2	96.4
Median (2 week)	2577	0.91	3771	63.1	92.9
Median (4 week)	2700	0.95	3725	70.2	92.9
Mean (6 week)	2772	0.98	3925	65.5	94.1
Untrained Median∗	2828	1.00	4103	70.2	91.7
Median (6 week)	3065	1.08	4215	64.3	91.7
Baseline	5025	1.78	6698	35.7	71.4
**State/Territory Ensemble, 22**–**23**					
Median (2 week)	54	0.87	83	47.1	87.3
Mean (2 week)	55	0.89	83	46.3	81.5
Median (4 week)	56	0.90	85	51.2	87.0
Mean (6 week)	56	0.91	87	45.4	85.4
Median (6 week)	57	0.92	89	49.8	88.4
Mean (4 week)	59	0.95	87	47.2	83.7
Untrained Median∗	62	1.00	93	46.3	81.2
Baseline	85	1.37	107	31.8	67.8
**State/Territory Ensemble, 23**–**24**					
Mean (6 week)	40	0.94	62	53.9	95.8
Median (6 week)	41	0.97	64	51.7	94.9
Median (2 week)	42	0.97	64	50.8	94.2
Mean (2 week)	42	0.98	64	52.1	94.9
Mean (4 week)	42	0.98	65	53.3	95.2
Untrained Median∗	43	1.00	66	50.7	92.1
Median (4 week)	44	1.03	69	51.4	94.3
Baseline	57	1.35	79	20.4	86.2
**State/Territory Ensemble, 24**–**25**					
Median (2 week)	72	0.96	110	57.4	93.5
Mean (2 week)	72	0.96	110	59.2	93.7
Untrained Median∗	75	1.00	110	61.3	91.4
Mean (4 week)	75	1.01	115	60.3	94.7
Median (6 week)	76	1.01	114	58.3	94.3
Median (4 week)	76	1.01	115	57.5	93.4
Mean (6 week)	78	1.04	117	61.1	94.9
Baseline	125	1.66	160	36.5	77.8

**Abbreviations:** MAE-mean absolute error, rWIS-relative weighted interval score, WIS- weighted interval score.

∗ The untrained median is the current operational FluSight-ensemble approach.

**Fig. 2:**
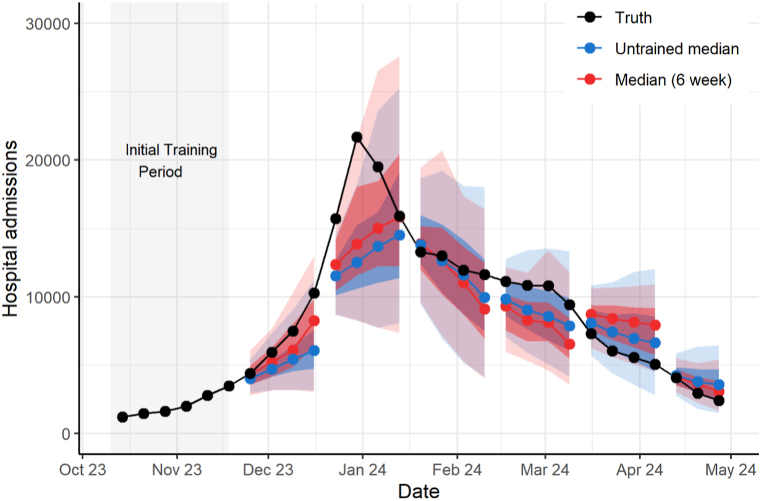
**National weekly observed hospital admissions and forecast distribution with 50% and 95% prediction intervals for the top performing trained ensemble (6-week median) and untrained ensembles during the 2023**–**24 influenza season.** We show each horizon (current and up to three weeks after) for each forecast. To avoid plotting overlapping forecasts, we only display ensemble forecasts every four weeks. The median ensemble forecast values (colored points) are shown with the corresponding 50% and 95% prediction intervals (colored shaded regions). Truth data were observed as of July 19, 2025.

For the 2023–2024 season, we used 30 different component forecasts to produce trained ensemble forecasts for 23 weeks. For a given week, the number of forecasts included ranged from 14 to 28 for the national ensemble and 11 to 19 for the state/territory ensemble. As with the 2022–2023 season, component forecast weight distribution varied throughout the season (Supplemental Fig. 2). For the national ensemble, the trained median and mean ensembles with 6-week training windows and the mean 2-week performed better than the untrained ensemble (rWIS 0.91, 0.98, and 0.99 respectively) (Table 1). All trained national ensembles, except the trained median with a 2-week training window, had 95% coverage that was the same or closer to 95% than the corresponding coverage from the untrained ensemble. During the period of increasing influenza hospital admissions (October through December), the median with a 6-week training window predicted more hospital admissions and was closer to the observed number of reported hospital admissions than the untrained median (Fig. 2). For the state/territory ensemble, the mean ensemble with a 6-week training window had the lowest rWIS of 0.94; all other trained ensembles except the median with a 4-week training window also performed better than the untrained ensemble. All trained state/territory ensembles had 95% coverage that was closer to 95% than that of the untrained ensemble.

**Fig. 3:**
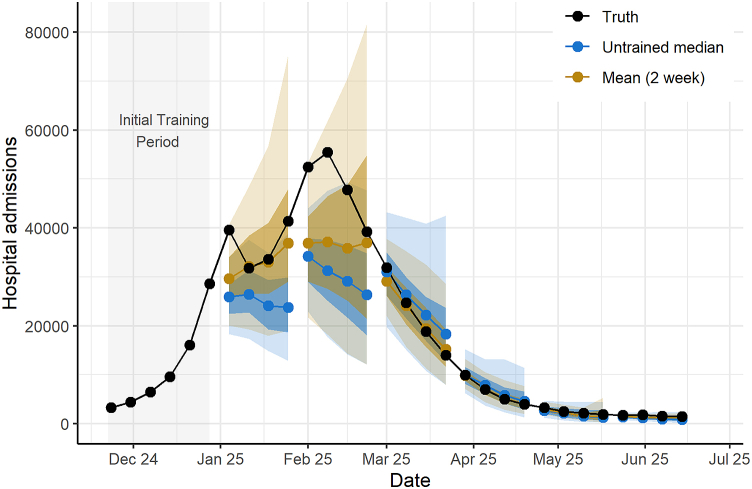
**National weekly observed hospital admissions and forecast distribution with 50% and 95% prediction intervals for the top performing trained ensemble (2-week mean) and untrained ensembles during the 2024**–**25 influenza season.** We show each horizon (current and up to three weeks after) for each forecast. To avoid plotting overlapping forecasts, we only display ensemble forecasts every four weeks. The median ensemble forecast values (colored points) are shown with the corresponding 50% and 95% prediction intervals (colored shaded regions). Truth data were observed as of June 19, 2025.

For the 2024–25 ensemble, we used 37 different component forecasts to produce trained ensemble forecasts for 21 weeks. For a given week, the number of forecasts included ranged from 16 to 31 for the national ensemble and 13 to 23 for the state/territory ensemble. Similarly to previous seasons, component forecast weight distribution varied throughout the evaluation period (Supplemental Fig. 3). For the national ensemble, all the trained mean ensembles and the 2- and 4-week median ensembles performed better than the untrained ensemble (Table 1). All of the national trained ensembles had 95% coverage equal to or better than the untrained ensemble. During the period of increasing influenza hospital admissions (January through February), the mean ensemble with a 2-week training window predicted more hospital admissions than the untrained median and was closer to the observed number of reported hospital admissions (Fig. 3). For the state/territory ensemble, the median and mean 2-week trained ensembles performed better than the untrained median, and the trained median with a 2-week training window performed the best (rWIS of 0.96). All trained state/territory ensembles also had 95% coverage that was closer to 95% than that of the untrained median. The relative WIS scores were closer than seen in other seasons, with a difference in range of 0.08 between the highest and lowest ranked trained ensembles, and only a 0.04 difference between the top performing model and the untrained median.

## 4 Discussion

For three influenza seasons, 2022–23 through 2024–25, we found that on average over an entire season, at least one trained ensemble of influenza hospital admissions forecasts had better performance than the untrained median ensemble at both the national and state levels. However, which trained ensemble that outperformed the untrained median ensemble varied by the national vs. state-level, season, and the forecast evaluation metric and included ensembles calculated using a mean or median approach and trained using different amounts of data. Across the first two seasons and between the national and state/territory level, the median ensemble with a 6-week training period consistently performed better than the untrained ensemble, albeit with sometimes marginal improvements. However, in the 2024–25 season, the 6-week median trained ensemble performed worse than the untrained ensemble at both the national and state level. This could be affected in part by the more dynamic nature of the 2024–25 season, which had multiple peaks and rapid changes in influenza hospital admissions. These findings could imply that seasons with greater variability may be better predicted by weighted ensembles that utilize shorter training windows, as this approach emphasizes models that are more accurate when evaluated against more recent trends. Comparatively, longer training periods may be better when there are more stable influenza trends that could result in more consistency in the set of top performing models, but ensembles based on these approaches may be slower to shift weights to forecast models that are better capturing the rapid changes. These findings are similar to the mixed success of trained ensemble methods from COVID-19 forecasting efforts where trained ensembles performed better when component forecasts had good and stable performance. Component forecast weights for the trained influenza ensembles varied considerably during the included influenza seasons, which might be more like COVID-19 case forecasting results, where the top performing models shifted over time and component forecast performance was inconsistent (Ray et al., 2023). This also may lead to the observed differences in trained ensemble performance across the influenza seasons included in this analysis and between the national and states/territory ensembles.

There are a few limitations to this work. First, because of the earlier timing of seasonal influenza during the 2022–23 in the United States and the start of the forecasting period, we were unable to compare all trained ensembles before the peak of the epidemic curve. Second, we were unable to use forecast performance during the earlier seasons to inform performance during the later seasons because submitted component forecast models differed from year to year (e.g., forecasts from 26 models were used for 2022–23,30 were used for 2023–24, and 35 for 2024–25). Operationally, this reliance on new training periods each season results in delays in implementation until sufficient weeks of training data are available, resulting in less ability to evaluate the performance of the trained ensembles early in the season. Additionally, we considered a small set of potential weighted ensemble models. Continued research on other weighted ensembling strategies is also needed. For example, this could include approaches that begin with equal weights and adjust gradually as evidence accumulates over the course of a season (McAndrew & Reich, 2021). In contrast, the weighting strategy considered here focuses on short term performance windows, which could lead to fluctuations in the distribution of model weights.

While weighted ensembles with different training periods often performed better than the untrained median ensemble (based on both WIS and coverage), there was no one specific weighted ensemble that consistently performed better. The stability of the untrained median ensemble, shown both for COVID-19 and influenza forecasting (Mathis et al., 2024; Ray et al., 2023) suggests it may be the preferred method of combining diverse influenza hospital admissions forecasts until more consistency in the performance of one or more trained ensembles is identified. As this is a limited analysis with only 3 influenza seasons, we will continue to evaluate ensemble performance over subsequent influenza seasons to see if the use of a specific trained ensemble performs better than the untrained median ensemble or whether adaptive approaches that update weighting strategies as the season evolves may provide improved performance.

## CRediT authorship contribution statement

**Aaron M. Frutos:** Writing – review & editing, Writing – original draft, Visualization, Formal analysis, Data curation, Conceptualization. **Annabella Hines:** Conceptualization, Data curation, Formal analysis, Visualization, Writing – review & editing. **Li Shandross:** Writing – review & editing, Conceptualization. **Evan L. Ray:** Writing – review & editing, Conceptualization. **Nicholas G. Reich:** Writing – review & editing, Conceptualization. **Rebecca K. Borchering:** Writing – review & editing, Data curation, Conceptualization. **Matthew Biggerstaff:** Writing – review & editing, Conceptualization.

## Disclaimer

The findings and conclusions in this report are those of the authors and do not necessarily represent the official position of the National Institutes of General Medical Sciences, the National Institutes of Health, or the Centers for Disease Control and Prevention.

## Funding

NGR, LS and ELR have been supported by the 10.13039/100000057National Institute of General Medical Sciences (R35GM119582) and the US 10.13039/100000030Centers for Disease Control and Prevention (U01IP001122).

## Declaration of competing interest

The authors declare that they have no known competing financial interests or personal relationships that could have appeared to influence the work reported in this paper.

## References

[bib1] About Flu Forecasting (2024). https://www.cdc.gov/flu-forecasting/about/index.html.

[bib2] Bracher J., Ray E.L., Gneiting T., Reich N.G. (2021). Evaluating epidemic forecasts in an interval format. PLoS Computational Biology.

[bib3] Centers for Disease Control and Prevention (2025). https://www.cdc.gov/flu-forecasting/evaluation/2023-2024-report.html.

[bib4] Centers for Disease Control and Prevention (2025). https://www.cdc.gov/flu-forecasting/evaluation/2024-2025-report.html.

[bib5] Cramer E.Y., Ray E.L., Lopez V.K., Bracher J., Brennen A., Castro Rivadeneira A.J., Gerding A., Gneiting T., House K.H., Huang Y., Jayawardena D., Kanji A.H., Khandelwal A., Le K., Muhlemann A., Niemi J., Shah A., Stark A., Wang Y., Reich N.G. (2022). Evaluation of individual and ensemble probabilistic forecasts of COVID-19 mortality in the United States. Proc Natl Acad Sci U S A.

[bib6] Flusight-forecast-data (2023). https://github.com/cdcepi/Flusight-forecast-data.

[bib7] FluSight-forecast-hub (2024). https://github.com/cdcepi/FluSight-forecast-hub.

[bib8] Hospital Respiratory Data (2024). https://www.cdc.gov/nhsn/psc/hospital-respiratory-reporting.html.

[bib9] Influenza Activity in the United States (2023). https://www.cdc.gov/flu/whats-new/22-23-summary-technical-report.html.

[bib10] Mathis S.M., Webber A.E., Leon T.M., Murray E.L., Sun M., White L.A., Brooks L.C., Green A., Hu A.J., McDonald D.J., Rosenfeld R., Shemetov D., Tibshirani R.J., Kandula S., Pei S., Shaman J., Yaari R., Yamana T.K., Agarwal P., Borchering R.K. (2024). Evaluation of FluSight influenza forecasting in the 2021-22 and 2022-23 seasons with a new target laboratory-confirmed influenza hospitalizations. Nature Communications.

[bib11] McAndrew T., Reich N.G. (2021). Adaptively stacking ensembles for influenza forecasting. Statistics in Medicine.

[bib12] Ray E.L., Brooks L.C., Bien J., Biggerstaff M., Bosse N.I., Bracher J., Cramer E.Y., Funk S., Gerding A., Johansson M.A., Rumack A., Wang Y., Zorn M., Tibshirani R.J., Reich N.G. (2023). Comparing trained and untrained probabilistic ensemble forecasts of COVID-19 cases and deaths in the United States. International Journal of Forecasting.

[bib13] scoringutils (2023). Scoringutils. https://github.com/epiforecasts/scoringutils.

[bib14] Shandross L., Howerton E., Contamin L., Hochheiser H., Krystalli A., Reich N.G., Ray E.L., Consortium of Infectious Disease Modeling Hubs (2026). Multi-model ensembles in infectious disease and public health: Methods, interpretation, and implementation in R. Statistics in Medicine.

